# Prediction of novel precursor miRNAs using a context-sensitive hidden Markov model (CSHMM)

**DOI:** 10.1186/1471-2105-11-S1-S29

**Published:** 2010-01-18

**Authors:** Sumeet Agarwal, Candida Vaz, Alok Bhattacharya, Ashwin Srinivasan

**Affiliations:** 1 Systems Biology Doctoral Training Centre and Department of Physics, University of Oxford, Clarendon Laboratory, Parks Road, Oxford OX1 3PU, UK; 2 Center for Computational Biology and Bioinformatics, School of Information Technology, Jawaharlal Nehru University, New Delhi 110067, India; 3 School of Life Sciences, Jawaharlal Nehru University, New Delhi 110067, India; 4 Oxford University Computing Laboratory, Wolfson Building, Parks Road, Oxford OX1 3QD, UK; 5 IBM India Research Lab, 4-C, Vasant Kunj Institutional Area Phase II, Vasant Kunj, New Delhi 110070, India; 6 School of Computer Science and Engineering & Centre for Health Informatics, University of New South Wales, Kensington, Sydney, Australia

## Abstract

**Background:**

It has been apparent in the last few years that small non coding RNAs (ncRNA) play a very significant role in biological regulation. Among these microRNAs (miRNAs), 22-23 nucleotide small regulatory RNAs, have been a major object of study as these have been found to be involved in some basic biological processes. So far about 706 miRNAs have been identified in humans alone. However, it is expected that there may be many more miRNAs encoded in the human genome. In this report, a "context-sensitive" Hidden Markov Model (CSHMM) to represent miRNA structures has been proposed and tested extensively. We also demonstrate how this model can be used in conjunction with filters as an *ab initio *method for miRNA identification.

**Results:**

The probabilities of the CSHMM model were estimated using known human miRNA sequences. A classifier for miRNAs based on the likelihood score of this "trained" CSHMM was evaluated by: (a) cross-validation estimates using known human sequences, (b) predictions on a dataset of known miRNAs, and (c) prediction on a dataset of non coding RNAs. The CSHMM is compared with two recently developed methods, miPred and CID-miRNA. The results suggest that the CSHMM performs better than these methods. In addition, the CSHMM was used in a pipeline that includes filters that check for the presence of EST matches and the presence of Drosha cutting sites. This pipeline was used to scan and identify potential miRNAs from the human chromosome 19. It was also used to identify novel miRNAs from small RNA sequences of human normal leukocytes obtained by the Deep sequencing (Solexa) methodology. A total of 49 and 308 novel miRNAs were predicted from chromosome 19 and from the small RNA sequences respectively.

**Conclusion:**

The results suggest that the CSHMM is likely to be a useful tool for miRNA discovery either for analysis of individual sequences or for genome scan. Our pipeline, consisting of a CSHMM and filters to reduce false positives shows promise as an approach for *ab initio *identification of novel miRNAs.

## Background

Several classes of small "non-coding RNA" (RNA sequences which are not translated to proteins) have been discovered in the last decade and have been found to play a central role in biological processes. One such class of non-coding RNA is microRNA (miRNA). Mature miRNA sequences are single stranded, typically 20-25 nucleotides long and encoded as a precursor molecule of about 60-120 nucleotides (in humans). These precursors are derived from processing of a pri-miRNA (usually in kilobases) by a ribonuclease, such as Drosha. Pre-miRNAs are also further cleaved to generate active mature miRNA with the help of Dicer.

Computational approaches to identify miRNAs are based on major properties of previously identified miRNAs, such as presence of a hairpin-shaped stem loop like secondary structure, evolutionary conservation and low minimum free energy. Most of these tools share the same overall strategy but use different approaches [1]. Some of the tools, such as **MiRscan **[2], use a filtering criteria to pick out pre-miRNAs from the initial set of candidate stem-loops based on GC content, minimum free energy and structural filters. This fails to identify all the known miRNAs with a high level of accuracy. "Homology-based" approaches exploit information from both sequence and structure to find new members of known miRNA families (homologous miRNAs) but cannot detect new miRNAs. Examples of these are profile based **ERPIN **[3] and **MiRAlign **[4]. **ProMiR **[5], a probabilistic co-learning method that relies on the paired HMM, models characteristics of the stem portion of the stem-loop viewed as a paired sequence. It uses a set of additional filters like comparison to other vertebrate genomes. A number of SVM-based machine learning methods have also been developed for prediction of miRNAs. **Triplet-SVM **[6] recognizes pre-miRNAs based on the presence of small (3 nt) structural features. SVM-based **MIRFinder **[7] was designed for analyzing genome-wide, pair-wise sequences from two related species and **RNAmicro **[8] uses twelve different features/descriptors, such as sequence composition, sequence conservation, structure, structure conservation and thermodynamic stability for SVM classification. It uses a preprocessor that identifies conserved 'almost-hairpins' in a multiple sequence alignment. **miPred**[9] uses a set of 29 features, consisting of global and intrinsic RNA folding measures, to construct an SVM classifier to distinguish between precursors and non-precursors.

Other kinds of learning-based prediction tools have also been developed, such as a random forest prediction technique **MiPred **[10] that uses a set of tree-based classifiers combining sampling of training data with random feature selection, and linear genetic programming-based **MiRPred **[11]. MiRPred uses 16 classifiers and an EST match filter. These tools generally use pairwise/multiple alignments for scanning, except for Triplet-SVM and MIRPred that use a single genome; and these have been evaluated on a single chromosome, or a part of a chromosome.

Hybrid approaches involving both experimentation and computation have also been used for large scale novel miRNA discovery. One such approach is to sequence small RNAs and then to analyse these in terms of known and novel miRNAs using miRNA prediction tools [12]. **miRDeep **uses a probabilistic, additive scoring method to detect miRNAs [13]. However, some of the filters used for scoring are highly stringent and likely to miss many miRNAs. This report describes a miRNA prediction method which uses a context-sensitive Hidden Markov Model (CSHMM) and examines its application for predicting new miRNAs in the human genome.

## Methods

### Datasets

The following datasets were used for experiments in this paper:

**(D1) **The primary and secondary structures of 323 human miRNA precursors (these were obtained from miRBase); **(D2) **The primary structure of 646 "pseudo-hairpin" sequences [9]; i.e., sequences from human genic regions which can fold up into a hairpin structure, similar to pre-miRNA. These are expected to contain no miRNA precursors; **(D3) **The primary structures of 1,918 non-human miRNA precursors from 40 different species (taken from the datasets used by Ng and Mishra [9]); **(D4) **The non coding RNA set (Ensembl). Homo_sapiens.NCBI36.54.ncrna.fa; **(D5) **Small RNA sequences obtained from normal human leukocytes.

### Cross validation and Hold out Tests

Part of datasets D1 and D2 (200 and 400 sequences respectively) were used as the training data (this was identical to that used by Ng and Mishra [9]) to construct the final classification tree. The remaining sequences from these two datasets, along with datasets D3 and D4 were used as test data on which predictions were made. To exclude the influence of same-family members on the test results, all human MiRNAs from a given text set which had a member of the same family in the training set were removed; additionally, for dataset D3, only one member of each family in a test set was kept. Thus the 123 remaining human precursors were purged of all the actual human pre-miRNAs belonging to families that were also represented in the training set (there were 41 of these). The residual test set comprised 82 human pre-miRNAs and 246 pseudo-hairpins. Similarly the Dataset D3 (1,918 non human miRNA sequences) was reduced to 512 sequences on removing family similarities. The known miRNAs were removed from the non coding RNA set D4 and the rest (6,978) were used as a test set as many of the other ncRNAs also form miRNA-like secondary structures. For details of the cross validation see Additional file 1.

### Representing miRNA precursors

**Figure 1:**
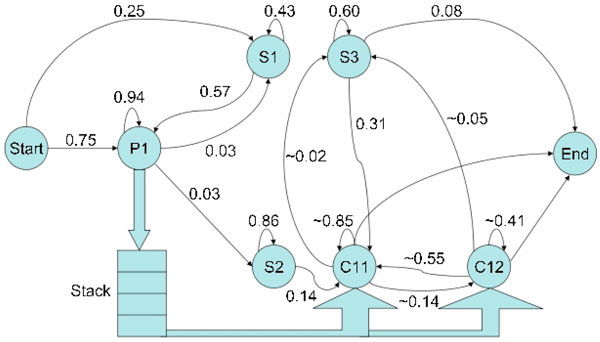
**The context-sensitive HMM proposed to represent miRNA precursors with estimated transition probabilities**. State P1 emits the upper halves of the stem and symmetric bulges. States S1 and S3 emit the asymmetric bulges in the upper and lower sections respectively. State S2 emits the loop. States C11 and C12 emit the lower halves of the stem and symmetric bulges respectively (~ refers to probabilities averaged over the four possible top-of-stack symbols).

Regular HMMs cannot be used to generate the language of miRNA precursors: ignoring the loop, this language is that of palindromes with distant interactions between nucleotides and we need at least a context-free grammar to represent it. However, the idea of CSHMMs has been recently proposed [14]. These are capable of representing such sequences. CSHMMs extend the idea of HMMs by introducing a memory, in the form of a stack or a queue, between certain states in the model. The original idea was to have a pairwise-emission state, which would put a copy of every symbol emitted by it into the associated memory, and a single corresponding context-sensitive state, which would read a symbol from the memory, and based on it, would then decide what to emit and where to transit. To represent miRNA structures, we have extended this idea slightly. The CSHMM structure we propose has two context sensitive states which are linked to the same pairwise-emission state through a stack. This is because we need separate states to generate the stem and the symmetric bulges; yet both these states need information about what was emitted earlier (the stem state, so that it may emit the complementary nucleotides; and the symmetric bulge state so that it may ensure the symmetry of the bulge). The structure of the CSHMM we propose is shown in Fig. 1. Here states labeled as P are pairwise-emission states, those labeled as C are context-sensitive ones, and those labeled as S are regular HMM states.

### Identifying miRNA precursors

#### Parameter estimation

A complete CSHMM consists not just of the structure, but also of probabilities for the symbols emitted and the probabilities of transition from one state to another (usually called emission and transition probabilities). Given data of known stem-loop structures, these probabilities can be estimated by keeping count of the different transition and emission events for all the states. With these counts, estimates of the emission and transition probabilities can be obtained using the following formulae [15]:(1)(2)

Here, *P*_*e *_is the probability of emitting symbol *σ *in state *q*; and *P*_*t *_the probability of transiting from state *q *to *q'*. *Q *is the set of all states in the models; Σ is the output alphabet, consisting in this case of A, C, G and U; *c*_*t *_and *c*_*e *_are the transition and emission counts obtained from the labeled data.

For the two context-sensitive states, the symbol at the top of the stack also has to be taken into account. Accordingly, we modify the formulae above as follows (here *α *represents a letter from the alphabet, *i.e*. A, C, G or U):(3)(4)

#### Discrimination

Given a complete CSHMM (structure and probabilities), and any input sequence, an optimal alignment algorithm for computing the most likely sequence of states using the CSHMM is known [16], We cannot, however, use this algorithm to discriminate between miRNA precursors and other kinds of RNA sequences. For each such sequence, the algorithm simply gives us two things: the most likely state sequence (and hence, secondary structure) and the likelihood of obtaining that state sequence. Nevertheless, if the parameters have been estimated using miRNA precursors, we can expect relatively high likelihoods for such sequences. In addition, we would also expect to see a much closer match between the true secondary structure of miRNA sequences and the structure predicted by the alignment algorithm.

In this paper, we investigate a very simple discriminatory function that uses the results from the alignment algorithm. For our model, discrimination is a function only of the likelihood returned by the alignment algorithm. The form of the discriminatory function is thus just a single-node classification tree [17], which corresponds to a threshold on the likelihood score. The value of this threshold is estimated from sequences of miRNA precursors and non-precursors. Each sequence is provided to the alignment algorithm, which uses the CSHMM from Stage 1 to return a likelihood value. A classification tree is then constructed to discriminate between the two sets of sequences, using just one feature: the likelihood value.

## Results and discussion

### Performance of the CSHMM-based miRNA classifier

The performance of the two-stage procedure for identifying miRNA precursors described here was assessed by: (a) cross-validation estimates of predictive performance, (b) predictions on an independent dataset of known miRNA precursors, and (c) prediction on a dataset of non coding RNAs. For comparison purposes, we also present the results obtained by using the recently described miPred classifier [9] on the same data. The datasets used here are described in further detail under Methods.

The final CSHMM structure, along with estimates of the transition probabilities, is shown in Fig. 1. Results from the classification tree model built using the CSHMM likelihood scores are presented here, alongside those obtained with miPred. The 5-fold cross-validation estimate of predictive performance for our model on the human RNA training data (600 sequences, 200 from Dataset D1 and 400 from Dataset D2) is in Table 1. The cross-validation was done such that the miRNAs belonging to the same family were kept in a single fold. For miPred, the authors do not report the details of the 5-fold cross-validation results; only the overall accuracy is mentioned as **93.5%**. Results on the test set (remaining sequences from D1 and D2) for the respective classifiers are in Table 2. The CSHMM-based classifier identified 94% of the total non-human miRNAs (Dataset D3) and 83% of the purged D3 set (without sequence similarity), and reported 4% of the non coding RNAs (Dataset D4) as miRNAs. The principal observations that we can make from the results are these:

**Table 1:** 5-fold cross-validation Performance of the CSHMM using a human miRNA dataset.

		Actual		
**Predicted**		**miRNA**	**non-miRNA**	
	
	miRNA	170(60.67)	12(121.33)	182
	
	non-miRNA	30(139.33)	388(278.67)	418
	
		200 (dataset D1)	400 (dataset D2)	600

The number in parentheses following each entry is the expected value of the entry under the hypothesis that the actual class is independent of the predicted one. Estimates of predictive accuracy, sensitivity and specificity from this table are 0.93 (93%), 0.85 (85%) and 0.97 (97%) respectively.

**Table 2:** Predictive performance of CSHMM and *miPred *on a common test dataset.

(a) CSHMM				
		**Actual**		

**Predicted**		**miRNA**	**non-miRNA**	
	
	miRNA	63(16.75)	4(50.25)	67
	
	non-miRNA	19(65.25)	242(195.75)	261
	
		82 (dataset D1)	246 (datasetD2)	328

**(b) *miPred***				

		**Actual**		

**Predicted**		**miRNA**	**non-miRNA**	
	
	miRNA	64(17.25)	5(51.75)	69
	
	non-miRNA	18(64.75)	241(194.25)	259
	
		82 (dataset D1)	246 (dataset D2)	328

The number in parentheses following each entry is the expected value of the entry under the hypothesis that the actual class is independent of the predicted one. Estimates of predictive accuracy, sensitivity and specificity of CSHMM (a) from this table are 0.930 (93.0%), 0.768 (76.8%), and 0.984 (98.4%) respectively. For *miPred *(b) these are 0.930 (93.0%), 0.780 (78.0%) and 0.980 (98.0%) respectively.

**Figure 2:**
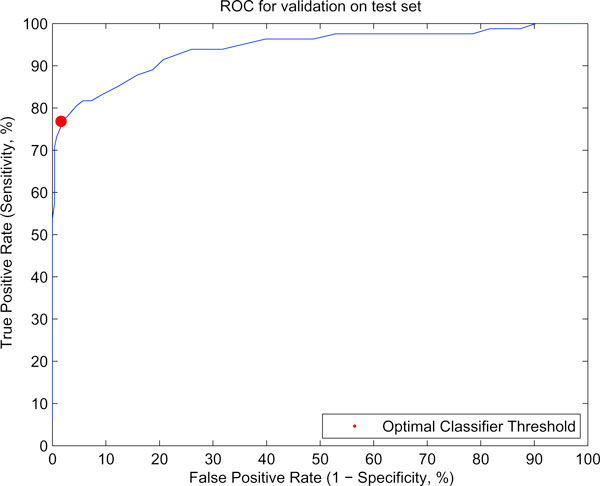
**Receiver-Operating Characteristic (ROC) curve for the CSHMM classifier on the test set**. Classification was done for a range of thresholds on the likelihood score, and true and false positive rates computed for each case. The point in red shows the results of the 'optimal' threshold, as determined by entropy minimization, and corresponds to the results reported in Table 2(a).

(1) The CSHMM-based classifier performs as well as the SVM-based model used by miPred: on both human and non-human pre-miRNA test sets, our model's results are as good as or slightly better than those of miPred. The primary advantage of the CSHMM is that it is a generative model, as opposed to a discriminative model like the SVM used by miPred. This means that not only can we use the CSHMM to identify likely pre-miRNA sequences, but can also use it to predict the most likely secondary structure for a given pre-miRNA candidate. The CSHMM specifies a probability distribution over all possible secondary structures.

(2) The test results are largely in agreement with the 5-fold cross-validation estimates of Table 1. In particular, we see that both sensitivity and specificity values obtained on the human pre-miRNA and "pseudo-hairpin" set (Table 2) are very similar to the cross-validation estimates. For nonhuman miRNAs, the sensitivity observed is about 94% in comparison to sensitivity of 92% obtained with miPredon the whole set (1,918 sequences). On removal of sequence similarities (leaving 512 sequences, as described in Methods) the sensitivity is 83%. We have also analysed other non coding RNAs (ncRNA, dataset D4) for checking the specificity of the CSHMM. Only 4% of the sequences were identified as miRNAs, suggesting that the method discriminates well between actual miRNAs and other ncRNAs. Thus, in essence, only one feature (the likelihood score from the CSHMM) is effectively capturing all of the structural information encapsulated in the set of 29 features used by the miPredclassifier. We do need to store all of the emission and transition probabilities, but these are parameters of the CSHMM model as a whole, not features of each individual sequence. Once the CSHMM model has been learnt, we only need to calculate one feature per sequence, which is the likelihood score from the alignment. Thus, the CSHMM method greatly reduces the dimension of the feature space representation as compared to miPred's SVM model: a key advantage of our model is that it offers a much simpler representation of miRNA precursors. Rather than looking to use a lot of different folding measures like thermodynamic free energy, entropy, dinucleotide frequency etc. to predict whether a sequence is a pre-miRNA or not, the CSHMM looks to statistically determine and encode the secondary structure features of actual miRNA precursors. By doing so, it not only allows us to make predictions on new sequences (based on a threshold on the likelihood score), but also provides the most likely secondary structure for any given sequence on the assumption that it is a pre-miRNA. The threshold used by the classification tree represents just one possible cutoff on the CSHMM's likelihood score (obtained, in this case, by a method of minimising entropy). More generally, the performance of classifiers with different thresholds (resulting in correspondingly different true and false positive rates) can be summarised by a ROC curve. This is shown for holdout validation in Fig. 2. The curve shows a steep step-like slope, which usually suggests a good classifier across a range of thresholds.

### Identification of novel miRNAs using the CSHMM-based classifier

We are mainly interested in the identification of novel miRNAs. To this end, the CSHMM-based classifier was used to scan the entire chromosome 19. The classifier identified 70 out of the 80 known miRNAs present on this chromosome (Additional file 2). Around 18,188 additional hairpins having high likelihood scores were taken as a candidate set and were subjected to post-prediction filters comprising checks for the presence of EST matches and Drosha cutting sites. 100% matches with untranslatable ESTs were shown by 2,528 hairpins, out of which 49 harbored Drosha cutting sites (Additional file 3; most, but not all of these 49 novel precursors were also predicted by CID-miRNA [18] and MiPred). Additional file 4 shows the sequences and the structures of these predicted novel miRNAs.

We also carried out analysis of small RNA sequences from normal human leukocytes (Dataset D5). Flanking sequences of small RNA reads originating from the intergenic and intronic regions of the human genome were extracted and were folded by the CSHMM, CID-miRNA and miRDeep (to identify the precursors/hairpins harboring these sRNAs). The sRNAs falling within the same hairpin were classified as IsomiRs and star sequences [12] and grouped into a family. IsomiRs are sRNAs that fall within the same precursor sequence predicted and which have the same sequence but vary by a few nucleotides from each other on account of alternative Dicer cutting. Star sequences are sRNA that also fall within the same hairpin but have a partially complementary sequence.

The member with the highest frequency (expression level) was deemed as a novel miRNA. The CSHMM identified 359 sRNAs falling within hairpins out of which 308 were novel miRNAs owing to their highest frequency in their respective family. This was found to be comparable to that obtained by CID-miRNA. Since miRDeep is likely to miss many valid miRNAs due to a number of stringent criteria, such as expression level, used for prediction of novel miRNAs, it is not surprising that it identified only 22 sRNAs falling in hairpins out of which 5 were novel miRNAs. The Additional file 5 shows 18 sRNAs (common among the three tools) grouped into families and their respective representative novel miRNAs. The Additional file 6 shows the sequences and the structures of the 5 representative novel miRNAs.

## Conclusion

Methods that can recognise miRNAs without the restriction of sequence homology can help to focus the experimental effort for unknown families of miRNAs. In this paper, we have investigated one such method. The recognition is achieved using a recently proposed extension to Hidden Markov Models, which allows the development of probabilistic variants of context-sensitive grammars, which may be better suited to represent efficiently the "language" of miRNA precursors. Specifically, we: (a) propose a context-sensitive Hidden Markov Model (CSHMM) for recognizing miRNA structures; (b) use known human miRNA sequences to estimate transition and emission probabilities for the CSHMM; (c) obtain the most likely secondary structure for a given sequence of nucleotides using the CSHMM; and (d) use the likelihood values from the output of the CSHMM to construct a recognizer (in the form of a classifier) for miRNAs. The results suggest that we are able to develop a very simple classifier that shows a sensitivity of about 85% along with a specificity of about 97-98% on human miRNA sequences. Although not trained using non-human sequences, the recogniser is able to identify a substantial proportion of a set of known miRNAs from 40 different non-human species; the true-positive rate on these is around 83%. In addition it can also differentiate miRNAs from other ncRNAs that form miRNA-like secondary structures. Mature miRNA derived from one of the predicted sequences was experimentally detected verifying the prediction (not shown). The CSHMM-based classifier constructed here is available as an applet online [19].

## Competing interests

The authors declare that they have no competing interests.

## Authors' contributions

SA developed and implemented the CSHMM, and conducted most of the computational experiments. CV ran some of the applications, analyzed the genomic and small RNA sequence data and organized the results. AB planned the experimental part and helped to write sections of the manuscript. AS conceptualized the computational aspects of the problem and supervised SA.

## Supplementary Material

Additional file 1 **Methodological details of CSHMM implementation and computational complexity estimation**. Click here for file

Additional file 2 **Analysis of known miRNAs on Chromosome 19**. This file contains the list of the known miRNAs present on chromosome 19. 70 of these were predicted by CSHMM. Click here for file

Additional file 3 **Novel predicted miRNAs on Chromosome 19**. This file contains 9 intergenic and 40 intronic novel miRNAs predicted by the CSHMM along with their respective CSHMM likelihood scores, EST matches, Drosha site prediction scores and MiPred scores. Click here for file

Additional file 4 **Secondary structures of the novel predicted miRNAs on Chromosome 19**. Click here for file

Additional file 5 **Novel miRNAs from Small RNA sequence analysis**. The sRNAs are grouped into their respective IsomiR families as and when present. The ones highlighted in blue are the representative novel miRNAs, identified on the basis of highest frequency within the family. Click here for file

Additional file 6 **Secondary structures of the 5 representative novel miRNA from the sRNA sequence data**. Click here for file
