# Supplementary material for: Prediction of novel precursor miRNAs using a context-sensitive hidden Markov model (CSHMM)
Source: BMC Bioinformatics. 2010 Jan 18;11(Suppl 1):S29. doi: 10.1186/1471-2105-11-S1-S29 (PMC3009500; doi:10.1186/1471-2105-11-S1-S29)
Supplement: Additional file 1 — Methodological details of CSHMM implementation and computational complexity estimation. [file 1471-2105-11-S1-S29-S1.pdf]

Additional Text on the Methodology of CSHMM

Representing miRNA precursors

Like all RNA sequences, miRNA precursors are a series of the nucleotides consisting of adenine (A), cytosine (C), guanine (G) and uracil (U). Only three kinds of base-pairings are possible; these are A-U, C-G and G-U. The section of the secondary structure consisting largely of paired bases is known as the "stem". The stem is not continuous; there are portions in between where unpaired nucleotides bulge out. These bulges may be either symmetric or asymmetric; in the latter case, there are one or more gaps on one side, denoted by dashes. At the end of the structure, there is a "loop". This is essentially a large bulge, with no pairing.

Regular HMMs cannot be used to generate the language of miRNA precursors: ignoring the loop, this language is that of palindromes with distant interactions between nucleotides and we need at least a context-free grammar to represent it. However, the idea of CSHMMs has been recently proposed [1]. These are capable of representing such sequences. CSHMMs extend the idea of HMMs by introducing a memory, in the form of a stack or a queue, between certain states in the model. The original idea was to have a pairwise-emission state, which would put a copy of every symbol emitted by it into the associated memory, and a single corresponding context-sensitive state, which would read a symbol from the memory, and based on it, would then decide what to emit and where to transit. To represent miRNA structures, we have extended this idea slightly. The CSHMM structure we propose has two context sensitive states which are linked to the same pairwise-emission state through a stack. This is because we need separate states to generate the stem and the symmetric bulges; yet both these states need information about what was emitted earlier (the

stem state, so that it may emit the complementary nucleotides; and the symmetric bulge state so that it may ensure the symmetry of the bulge). The structure of the CSHMM we propose is shown in Fig. 1. Here states labeled as P are pairwise-emission states, those labeled as C are context-sensitive ones, and those labeled as S are regular HMM states. The state sequence corresponding to the secondary structure shown is as follows:

$$\textbf{Start} - (\textbf{P1})^{5}\,(\textbf{S1})(\textbf{P1})^{21}\,(\textbf{S2})^{27}\,(\textbf{C11})^{16}\,(\textbf{C12})^{2}\,(\textbf{C11})^{8} - \textbf{End}$$

Identifying miRNA precursors

Parameter Estimation

A complete CSHMM consists not just of the structure, but also of probabilities for the symbols emitted and the probabilities of transition from one state to another (usually called emission and transition probabilities). Given data of known stem-loop structures, these probabilities can be estimated by keeping count of the different transition and emission events for all the states. With these counts, estimates of the emission and transition probabilities can be obtained using the following formulae [2]

$$P_e\,(q,\sigma) = \frac{c_e(q,\sigma)}{\Sigma_{\rho\in\Sigma}\, c_e(q,\rho)} \qquad (1)$$

$$P_t\,(q,q') = \frac{c_t(q,q')}{\Sigma_{s\in Q}\, c_t(q,s)} \qquad (2)$$

Here, P_e is the probability of emitting symbol σ in state q; and P_t the probability of transiting from state q to q' . Q is the set of all states in the models; Σ is the output alphabet, consisting in this case of A, C, G and U; c_t and c_e are the transition and emission counts obtained from the labeled data.

For the two context-sensitive states, the symbol at the top of the stack also has to be taken into account. Accordingly, we modify the formulae above as follows (here α represents a letter from the alphabet, *i.e.* A, C, G or U):

$$P_e (q , \sigma \mid \alpha) = \frac{c_e (q , \sigma \mid \alpha)}{\Sigma_{\rho\in \Sigma}\, c_e (q , \rho \mid \alpha)} \quad (3)$$

$$P_t (q , q' \mid \alpha) = \frac{c_t (q , q' \mid \alpha)}{\Sigma_{s\in Q}\, c_t (q , s \mid \alpha)} \quad (4)$$

Computational Efficiency

The main time-consuming step in the above procedure is the alignment of each RNA sequence to the CSHMM. The time complexity of the original CSHMM alignment algorithm is given by [3]:

$$O (L^3 M_1^2 M) + O(L^2 M_1 M^2) + O(L^2 M_2^2 M) \quad (5)$$

Here L is the length of the sequence, M_1 is the number of pairwise-emission/context-sensitive states, M_2 is the number of single-emission states and M is the total number of states. In order to derive the specific complexity for our model we can insert the actual values for the latter 3 variables. However, we have two context-sensitive (C) states linked to a single pairwise-emission (P) state, and so do not have a single value for M_1 . We have to look at what each term represents in the original analysis [3].

The first term in (5) arises from considering pairs of unlinked P and C states (i.e., pairs that do not share the same memory store). Since our model contains no such pairs, this term vanishes altogether. The second term is from looking at pairs of linked P and C states: as can be seen from Fig. 1, we have two such pairs, (P 1, C11) and (P

1, C12). So $M_1 = 2$ here. Also, $M_2 = 3$ and $M = 8$. Thus, the time complexity T for aligning a RNA sequence of length L to our model is:

$$T(L) = O(L^2 \times 2 \times 64) + O(L^2 \times 9 \times 8) = O(L^2) \qquad (6)$$

This compares favourably to the alignment complexity for any given SCFG or CM, which would be $O(L^3)$. For a covariance model, the multiplying factor would just be the number of states M , which might be considerably less than our factor of $2 \times 64 \times 9 \times 8 = 200$: but even if it had just 6 states (which is the number of different types of typical CM states), the time complexity of our model would be less for all sequences of

length greater than $200/6 \approx 33$. As mentioned earlier, the miRNA precursors we are modelling here are between 60 and 120 nucleotides long in humans.

Discrimination

Given a complete CSHMM (structure and probabilities), and any input sequence, an optimal alignment algorithm for computing the most likely sequence of states using the CSHMM is known [4]. We cannot, however, use this algorithm to discriminate between miRNA precursors and other kinds of RNA sequences. For each such sequence, the algorithm simply gives us two things: the most likely state sequence (and hence, secondary structure) and the likelihood of obtaining that state sequence. Neverthless, if the parameters have been estimated using miRNA precursors, we can expect relatively high likelihoods for such sequences. In addition, we would also expect to see a much closer match between the true secondary structure of miRNA sequences and the structure predicted by the alignment algorithm.

In this paper, we investigate a very simple discriminatory function that uses the results from the alignment algorithm. For our model, discrimination is a function only of the likelihood returned by the alignment algorithm. The form of the discriminatory

function is thus just a single-node classification tree [5], which corresponds to a threshold on the likelihood score. The value of this threshold is estimated from sequences of miRNA precursors and non-precursors. Each sequence is provided to the alignment algorithm, which uses the CSHMM from Stage 1 to return a likelihood value. A classification tree is then constructed to discriminate between the two sets of sequences, using just one feature: the likelihood value. The utility of using this threshold on the CSHMM's likelihood score for identifying miRNA precursors is assessed empirically in the next section.

Baseline

In order to get a measure of the utility of using a CSHMM to model pre-miRNA secondary structure, we compared its performance in discriminating between miRNA precursors and non-precursors with the current best known program used. The fact that the model proposed by us has two context-sensitive states linked with a single pairwise emission state requires a slight modification to this algorithm. Discrimination would be easy if we had two CSHMMs: one for miRNA precursors and one for non precursors. For any input sequence, we could then simply classify it as one or the other based on the likelihood from each model. However, it is not clear how such a "non-precursor" model could be built, as these sequences do not have any definite structural properties for classifying pre-miRNAs. *miPred* [6] uses a set of 29 features, consisting of global and intrinsic RNA folding measures, to construct a Support Vector Machine (SVM) classifier to distinguish between precursors and non-precursors. Several of the features are derived from RNAfold [7, 8]. Other features are based simply on the sequence composition, e.g. dinucleotide frequencies.

Algorithms

The implementation of the algorithms for parameter estimation and optimal alignment was done by us following described methods. The parameter estimation program accepts a sequence of bases and the associated secondary structure and returns estimates of emission and transition probabilities. The optimal alignment program accepts a sequence of bases and returns the most likely secondary structure and its likelihood. The machine learning toolkit WEKA [9, 10] was used to construct the single-node classification tree for discriminating amongst miRNA precursors and non precursors. The tree is constructed using the J48 classification tree model builder provided in WEKA with default settings for the two principal parameters (C = 0.25,M = 2). The *miPred* results are taken directly from the paper by Ng and Mishra [6]. It was ensured that our training and test sequences are the same as those used by the authors, so that the results are comparable.

Cross-validation

The following k-fold cross-validation design to estimate predictive performance was used:

(1) Let T denote the dataset comprised of sequences from datasets D1 and D2 (described earlier in Methods section). Randomly partition T into k (near) equal parts $T_1, T_2, ..., T_{k.}$

(2) For i = 1 to k

(a) Let $Train_i$ consist of all the sequences in T_1, T_2, ..., T_{i-1}, T_{i+1}, ..., Tk ; and $Test_i$ consist of all the sequences in $T_{i.}$

(b) The parameters for the CSHMM in Fig. 1 using was estimated using the primary and secondary structures of miRNA precursors in $Train_{i.}$

(c) For each sequence in $Train_{i:}$ the trained CSHMM and the optimal

alignment algorithm were used to obtain the likelihood score.

(d) A classification tree was constructed (*Tree*$_i$), using the likelihood score only. This results in a simple threshold on the likelihood score being detected automatically.

(e) *Tree*$_i$ was used to predict the class (miRNA precursor or non-precursor) of sequences in *Test*$_i$. The predictions will result in some numbers of true positives (miRNA precursors correctly predicted as precursors), false positives (non-precursors predicted as precursors) false negatives (precursors predicted as non-precursors) and true negatives (non-precursors predicted as non - precursors).

The value of k used was 5.

(3) The primary and secondary structures of miRNA precursors in the complete dataset T was used to (re-)estimate the parameters of the CSHMM. This CSHMM, the optimal alignment algorithm, all the sequences in T, the feature extractor, and the classification tree-builder were used to construct a classification tree *Tree*.

(4) The counts of true positives, false positives, false negatives and true negatives from each of the *Tree*$_i$ predictions in Step 2e were summed. The resulting table provides a nearly unbiased estimate of the overall accuracy of prediction of tree *Tree* (the ratio of the number of correct predictions to the total number of sequences in T), as well as its overall true-positive rate or sensitivity (the ratio of true positives to the total number of miRNA precursors in T) and specificity (the ratio of true negatives to the total number of non-precursors in T).

References

[1] Yoon B-J and Vaidyanathan PP : **RNA secondary structure prediction using context-sensitive hidden Markov models**. In *Proceedings of IEEE International Workshop on Biomedical Circuits and Systems (BioCAS): Dec. 2004; Singapore.* IEEE, Piscataway, NJ, S2.7.INV-1-S2.7.INV-4.

[2] Seymore K, McCallum A, and Rosenfeld R: **Learning hidden Markov model structure for information extraction**. In *Proceedings of the AAAI'99 Workshop on Machine Learning for Information Extraction: July 1999; Orlando, FL.*

[3] Yoon, B.J., Vaidyanathan, P.P: **Context-sensitive hidden Markov models for modeling long range dependencies in symbol sequences**. *IEEE Transactions on Signal Processing 2006,* **54** (11) 4169–4184.

[4] Yoon B-J and Vaidyanathan PP : **Optimal alignment algorithm for context-sensitive hidden Markov models**. In *Proceedings of the 30th IEEE International Conference on Acoustics, Speech and Signal Processing: Mar. 2005; Philadelphia, PA.*

[5] Breiman L, Friedman J H, Olshen R A, and Stone C J: *Classification and Regression Trees (CART).*Wadsworth, Pacific Grove, CA; 1984.

[6] Ng KL, Mishra SK: **De novo SVM classification of precursor microRNAs from genomic pseudo hairpins using global and intrinsic folding measures**. Bioinformatics 2007, **23**(11):1321–1330.

[7] Hofacker IL, Fontana W, Stadler PF, Bonhoeffer S, Tacker M, Schuster P: **Fast folding and comparison of RNA secondary structures.** *Monatshefte für Chemie* 1994, **125:**167-188.

[8] Hofacker IL: **Vienna RNA secondary structure server.** *Nucleic Acids Res*

2003, **31**(13):3429-3431**.**

[9] Witten IH and Frank E: *Data Mining: Practical Machine Learning Tools and Techniques* (Second Edition). Morgan Kaufmann : San Francisco, CA; 2005.

[10] **Weka software** [http://www.cs.waikato.ac.nz/˜ml/weka/]
