# Supplementary material for: Prediction of novel precursor miRNAs using a context-sensitive hidden Markov model (CSHMM)
Source: BMC Bioinformatics. 2010 Jan 18;11(Suppl 1):S29. doi: 10.1186/1471-2105-11-S1-S29 (PMC3009500; doi:10.1186/1471-2105-11-S1-S29)
Supplement: Additional file 2 — Analysis of known miRNAs on Chromosome 19. This file contains the list of the known miRNAs present on chromosome 19. 70 of these were predicted by CSHMM. [file 1471-2105-11-S1-S29-S2.pdf]

Identification of known miRNAs found in Chromosome 19 by CSHMM

INTERGENIC miRNAs		strand	CSHMM Likelihood score
**A. intergenic region: NT_011295 [5205847..5213046]**			
1	hsa-mir-24-2 (5209903..5209975)	-	0.246
2	hsa-mir-27a (5210056..5210133)	-	0.250
3	hsa-mir-23a (5210203..5210275)	-	0.252
**B. intergenic region: NT_011295 [5214146..5250751]**			
1	hsa-mir-181c (5248315..5248424)	+	0.241
2	hsa-mir-181d (5248491..5248627)	+	0.238
**C. intergenic region: NT_011295 [10732366.. 10832202]**			
1	hsa-mir-640 (10808674..10808769)	+	-
**D. intergenic region: NT_011109 [18790093..18790658]**			
1	hsa-mir-769 (18790408..18790525)	+	0.262
**E. intergenic region: NT_011109 [24418323..24464969]**			
1	hsa-mir-99b (24464055..24464124)	+	0.245
2	hsa-let-7e (24464229..24464307)	+	0.260
3	hsa-mir-125a (24464697..24464782)	+	0.250
**F. intergenic region: NT_011109 [26408454..26533648]**			
1	hsa-mir-512-1 (26438123..26438206)	+	0.280
2	hsa-mir-512-2 (26440601..26440698)	+	0.269
3	hsa-mir-1323 (26443412..26443484)	+	0.276
4	hsa-mir-498 (26445641..26445764)	+	0.268
5	hsa-mir-520e (26447155..26447241)	+	0.291
6	hsa-mir-515-1 (26450447..26450529)	+	0.280
7	hsa-mir-519e (26451384..26451467)	+	0.276
8	hsa-mir-520f (26453603..26453689)	+	0.295

**9**	**hsa-mir-515-2 (26456453..26456535)**	+	**0.281**
**10**	**hsa-mir-519c (26457913..26457999)**	+	**0.287**
**11**	**hsa-mir-1283-1 (26459925..26460011)**	+	**0.276**
**12**	**hsa-mir-520a (26462325..26462409)**	+	**0.291**
**13**	**hsa-mir-526b (26465837..26465919)**	+	**0.288**
**14**	**hsa-mir-519b (26466657..26466737)**	+	**0.280**
**15**	**hsa-mir-525 (26468977..26469061)**	+	**0.270**
**16**	**hsa-mir-523 (26469829..26469915)**	+	**0.284**
**17**	**hsa-mir-518f (26471459..26471545)**	+	**0.276**
**18**	**hsa-mir-520b (26472671..26472731)**	+	**0.284**
**19**	**hsa-mir-518b (26474181..26474263)**	+	**0.268**
**20**	**hsa-mir-526a-1 (26477696..26477780)**	+	**0.290**
**21**	**hsa-mir-520c (26478897..26478983)**	+	**0.291**
**22**	**hsa-mir-518c (26480179..26480279)**	+	**0.280**
**23**	**hsa-mir-524 (26482446..26482532)**	+	**0.275**
**24**	**hsa-mir-517a (26483712..26483798)**	+	**0.279**
**25**	**hsa-mir-519d (26484791..26484878)**	+	**0.268**
**26**	**hsa-mir-521-2 (26488038..26488124)**	+	**0.263**
**27**	**hsa-mir-520d (26491540..26491626)**	+	**0.275**
**28**	**hsa-mir-517b (26492520..26492586)**	+	**0.260**
**29**	**hsa-mir-520g (26493610..26493699)**	+	**0.278**
**30**	**hsa-mir-516b-2 (26496886..26496970)**	+	**0.268**
**31**	**hsa-mir-526a-2 (26498366..26498430)**	+	**0.263**
**32**	**hsa-mir-518e (26501282..26501369)**	+	**0.292**
**33**	**hsa-mir-518a-1 (26502450..26502534)**	+	**0.273**
**34**	**hsa-mir-518d (26506321..26506407)**	+	**0.273**
**35**	**hsa-mir-516b-1 (26508289..26508378)**	+	**0.272**
**36**	**hsa-mir-518a-2 (26510777..26510863)**	+	**0.283**
**37**	**hsa-mir-517c (26512757..26512851)**	+	**0.279**
**38**	**hsa-mir-520h (26513956..26514043)**	+	**0.260**
**39**	**hsa-mir-521-1 (26520080..26520166)**	+	**0.259**
**40**	**hsa-mir-522 (26522655..26522741)**	+	**0.267**
**41**	**hsa-mir-519a-1 (26523841..26523925)**	+	**0.285**
**42**	**hsa-mir-527 (26525462..26525546)**	+	**0.279**
**43**	**hsa-mir-516a-1 (26528185..26528274)**	+	**0.284**

**44**	**hsa-mir-1283-2 (26529676..26529762)**	**+**	**0.277**
**45**	**hsa-mir-516a-2 (26532577..26532666)**	**+**	**0.275**
**G. intergenic region: NT_011109 [26547333..26565027]**			
**1**	**hsa-mir-371 (26559119..26559185)**	**+**	**0.275**
**2**	**hsa-mir-372 (26559334..26559400)**	**+**	**0.245**
**3**	**hsa-mir-373 (26560149..26560217)**	**+**	**0.267**
**H. intergenic region: NT_011109 [30287717..30306882]**			
**1**	**hsa-mir-1274b (30292565..30292631)**	**-**	**-**
	**INTRONIC miRNAs**		
	**NT_011255**		
**1**	**hsa-mir-1302-2 (11973 ..12110)**	**+**	**-**
**2**	**hsa-mir-1909 (1756158..1756237)**	**-**	**0.234**
**3**	**hsa-mir-1227 (2174061..2174148)**	**-**	**-**
**4**	**hsa-mir-637 (3901412..3901510)**	**-**	**0.230**
**5**	**hsa-mir-7-3 (4710682..4710791)**	**+**	**0.245**
**6**	**hsa-mir-220b (6435959..6436045)**	**+**	**0.232**
	**NT_011295**		
**1**	**hsa-mir-1181 (1776936..1777016)**	**-**	**-**
**2**	**hsa-mir-1238 (1925600..1925682)**	**+**	**-**
**3**	**hsa-mir-638 (2091882..2091981)**	**+**	**0.240**
**4**	**hsa-mir-199a-1 (2190904..2190974)**	**-**	**0.245**
**5**	**hsa-mir-639 (5903157..5903254)**	**+**	**0.228**
**6**	**hsa-mir-1470 (6823161..6823221)**	**+**	**0.236**
**7**	**hsa-mir-1270 (11772882..11772964)**	**-**	**0.263**
	**NT_011109**		
**1**	**hsa-mir-641 (13056668..13056766)**	**-**	**0.304**
**2**	**hsa-mir-330 (18410470..18410563)**	**-**	**-**
**3**	**hsa-mir-642 (18446404..18446500)**	**+**	**0.325**
**4**	**hsa-mir-220c (21331719..21331801)**	**-**	**-**
**5**	**hsa-mir-150 (22272232.. 22272315)**	**-**	**0.247**
**6**	**hsa-mir-643 (25053240..25053336)**	**+**	**0.287**

**7**	**hsa-mir-519a-2 (26533788..26533874)**	**+**	**-**
**8**	**hsa-mir-935 (26753751..26753841)**	**+**	**-**
