# Supplementary material for: Prediction of novel precursor miRNAs using a context-sensitive hidden Markov model (CSHMM)
Source: BMC Bioinformatics. 2010 Jan 18;11(Suppl 1):S29. doi: 10.1186/1471-2105-11-S1-S29 (PMC3009500; doi:10.1186/1471-2105-11-S1-S29)
Supplement: Additional file 4 — Secondary structures of the novel predicted miRNAs on Chromosome 19. [file 1471-2105-11-S1-S29-S4.pdf]

```
1)ACTGGATATGTACGTGTATGTGCACAGACACACCTTTGTGTGTGACCTGTGTGCAATCATATGCACATATATT
 C  G  A    C   TA         ---      C
A TG AT TGTA GTG  TGTGCACAG   ACACAC T
| || || |||| |||  |||||||||   ||||||
T AT TA ACGT TAC  ACGTGTGTC   TGTGTG T
 T  A  C    A   TA         CAG      T

2)GGTGGAGGCAGTTGGGTCGCGGCCCTGCCACCCCCATGCTGCACGGCCTCGGCCCAGTGCCACCACC
     A    G        C    C   CACCC
GGTGG GGCA TTGGGTCG GGCC TGC     C
||||| |||| |||||||| |||| |||
CCACC CCGT GACCCGGC CCGG ACG     C
     A    -        T    C   TCGTA

3)CCTGGGCAGAGTCTCATCTGTTAATGGGAGCTGGTCTAAGGGTCCTTCCAGCTCCCAGATTGATGATTTCCCAGG
      C  AGTC     T   AA          TCT
CCTGGG AG    TCATC GTT  TGGGAGCTGG   AAGGG
|||||| ||    ||||| |||  ||||||||||   ||||
GGACCC TT    AGTAG TAG  ACCCTCGACC   TTCCT
      T  ----     T   --          ---

4)GGCGAGGCGGGCCGAGACCCTTGGCTCGGGGCATTCAAGGGGAGTCCCCCCATGAACTGCGCCAAGCCTGGGTCCCGGGTG
GCCCAGCC
   GA   GGG   A     TT    C  G   -    A    A
GGC  GGC   CCG GACCC  GGCT GG GCA TTCA GGGG G
|||  |||   ||| |||||  |||| || ||| |||| |||| |T
CCG  CCG   GGC CTGGG  CCGA CC CGT AAGT CCCC C
   AC   GTG   C     T-    A  G   C    A    C

5)GGGCCCGGGGATGGGTCTGGGGGAGGCACTTCTGAGAAAAGGTGGAAACGGTTCTCAGAAACCCCGAAACCCTGAGGGACT
CTTTTCCTTCTCAGAAATGCTCCCCCCGCCCCAGACTCGGGAAA
GGG      GA    T T       G  C          AAGGT     C  T    AGAAACCC
   CCCGGG  TGGG C GGGGGAG CA TTCTGAGAA     GGAAA GG TCTC        C
   ||||||  |||| | ||||||| || |||||||||     ||||| || ||||        G
   GGGCTC  ACCC G CCCCCTC GT AAGACTCTT     CCTTT CT AGGG        A
AAA      AG    C C       -  A          -----     T  C    AGTCCCAA

6)AGCCCGGGGCAGCCATGGGGCCATCCACGCTGCTCTGACGGAGTGGCATCAGGAGCAGCGTGCATGCCCCACCTCCCTGGG
GT
 G       CA CCA      C  C          TGACGGA
A CCCGGGG  G   TGGGGC AT CACGCTGCTC       G
| |||||||  |   |||||| || ||||||||||       T
T GGGTCCC  C   ACCCCG TA GTGCGACGAG       G
 G       TC ---      -  C          GACTACG

7)ATTGTGTTTATGAGGCATTCTCGTTCTATGGAAACAAGGAATGGGATCGCCTGTCACCATTCACACAGT
       TT   -----    AT         A  G
ATTGTGT  ATG     AGGC  TCTCGTTCT TG A
|||||||  |||     ||||  ||||||||| ||
TGACACA  TAC     TCCG  AGGGTAAGG AC A
       CT   CACTG    CT         A  A
```

```
8)TCTATTTCTTTCTCTGTTTTATTATCTCATTCTTGGCTGTGAGGTAAACAGGAGAAAATGA
  TA      TCT      TATT      TTCT
TC  TTTCTT   CTGTTT    ATCTCA    T
||  ||||||   ||||||    ||||||
AG  AAAGAG   GACAAA    TGGAGT    G
  TA      ---      ----      GTCG

9)CAGCCCAGCGTCCCTGATTGGATAACTTCTAAGGTCCTGCCCCCGCAAACCCTGAATGAAAGATGTGATCAGACGCTGGGC
TG
           CC       G  AA   C  ---   TCC   C
CAGCCCAGCGT  CTGATTG AT  CTT TA   AGG   TGC C
|||||||||||  ||||||| ||  ||| ||   |||   |||
GTCGGGTCGCA  GACTAGT TA  GAA GT   TCC   ACG C
           --       G  --   A  AAG   CAA   C


10)CCCACCACTCTAACACTAAAGGCAGGAGTTCTGTTTGTGATATGACTCTGCCTTTATAGTGGGGGCTCCTGTGGG
     ---CA    A  AC          AGTTCTGTT
CCCAC     CTCT AC  TAAAGGCAGG         T
|||||     |||| ||  ||||||||||
GGGTG     GGGG TG  ATTTCCGTCT         G
     TCCTC    G  AT          CAGTATAGT


11)CTGGGTCTTGGCTGTACAGACCTCTGGGGCCCAGGTCTCCGGTATGGGACCTTTGACAGCCGAGACTTAG
               A    CCTCTG  G    G
CTGGGTCTTGGCTGT CAGA      GG CCCA GTCT
||||||||||||||| ||||      || |||| |||C
GATTCAGAGCCGACA GTTT      CC GGGT TGGC
               -    ------  A    A


12)CCTGTGGGCCCAGACCTTTCCCCTTCACACCTATCCATACCAGTGGGCCCAGGAGTGAGGAAGAAGGGGCTGGAACCCAC
TGG
  T     -C    A      CC      A   ATC A
CC GTGGG  CCAG CCTTTC  CTTCAC CCT   C TACC
|| |||||  |||| ||||||  |||||| |||   | |||
GG CACCC  GGTC GGGAAG  GGAGTG GGA   G GTGA
  T     AA    G      AA      A   CCC G


13)AGCACGGGGATGTAGATAGGCACACAGTTCACCTGGCCGTTCTCTGTTGCTGTCTGTCCCTGTCCACATCCTTCCCTGTG
CT
          ----   A      C   CAGTTCACCTGGCC
AGCACGGGGA    TGT GATAGG ACA              G
||||||||||    ||| |||||| |||
TCGTGTCCCT    ACA CTGTCC TGT              T
          TCCT   C      C   CTGTCGTTGTCTCT


14)GCCCGCCCCAGGAGGAGGCCCAAGGCTGCTCTCCCTCCTTTGGGACGTCACCAGCCTCCTCCTGGGCAG
GCC GC             CCAA   TG   TCCC
   C  CCCAGGAGGAGGC    GGC  CTC    T
   |  |||||||||||||    |||  |||
   G  GGGTCCTCCTCCG    CTG  GGG    C
--- AC             ACCA   CA   TTTC
```

```
15)CATGCCAGGCTGTTTGGCCTCAGGGAAGCAGCTTCCCTGACCAGTCCTTAGCACCTGGCACG
 A       CTG TT  C ---
C TGCCAGG   T  GG C   TCAGGGAAGC
| |||||||   |  || |   ||||||||||A
G ACGGTCC   A  CC G   AGTCCCTTCG
 C       ACG TT  T ACC
```

```
16)GGTCACCTCTCTGGCCGTCTACCTTCCACACTGACAAGGGCCGTGGGGACGTAGCTGGCCAGACAGGTGACC
        C        T    C    A   T AC
GGTCACCT TCTGGCCG CTAC TTCC CAC G  A
|||||||| |||||||| |||| |||| ||| |  A
CCAGTGGA AGACCGGT GATG AGGG GTG C  G
        C        C    C    -   C GG
```

```
17)TGACGTTCAGCTGCTGCCCCATCTGGAGTGACTGGAAGGACACTCTGGGGCAACACTGAGCGCCA
  A       C  C       TCTG     ACTG
TG CGTTCAG TG TGCCCCA    GAGTG    G
|| ||||||| || |||||||    |||||
AC GCGAGTC AC ACGGGGT    CTCAC    A
  C       -  A       ----     AGGA
```

```
18)GCCTCTGCTCGTGAGATGAGGGAAGCCTAGGCAGTACAGGCTCCCTGGCTTCTTTAATCTAGAGGAGGGGT
      G   GTG    G          TA  CAGT
GCCTCT CTC   AGAT AGGGAAGCC  GG    A
|||||| |||   |||| |||||||||  ||    C
TGGGGA GAG   TCTA TTTCTTCGG  CC    A
      G   A--    A          TC  TCGG
```

```
19)TTCTGCCCCATCAATTTCTAGATCAGGAAGGGGTTTGGAATCCAACACTCCTTCCCATTTAAGATGAGAAAGTGGAA
      CCCA   ATT   A  TCA         -
TTCTGC    TCA   TCT GA   GGAAGGGGT TTGGA
||||||    |||   ||| ||   ||||||||| |||||A
AAGGTG    AGT   AGA TT   CCTTCCTCA AACCT
      AAAG   ---   A  TAC         C
```

```
20)GTGAGGAGCCACGGGGACAGCATCCTGCAGATGGTCCTGGCCCTTGTCCCACCGACCTGTCTACAAGGACTGTCCTCGTG
GACCCTCCC
 T    AG            CA   -  C      TC --   CCC
G GAGG  CCACGGGGACAG  TCC TG AGATGG  C  TGG   T
| ||||  ||||||||||||  ||| || ||||||  |  |||
C CTCC  GGTGCTCCTGTC  AGG AC TCTGTC  G  ACC   T
 C    CA            --   A  A      CA CC   CTG
```

```
21)CCCTCTTGACTCTGGACCCCAAAATCTGGACCTCCAATGAAGCTGTCCCTTTGGGACTCCAGAATCCAGAGAG
 C    T  C      -C      ATCTGGACCTCC
C CTCT GA TCTGGA  CCCAAA            A
| |||| || ||||||  ||||||
G GAGA CT AGACCT  GGGTTT            A
 A    C  A      CA      CCCTGTCGAAGT
```

```
22)AGGCAGGAGCCGGCTTAGAGCCTCGGTTTCTCCACCTGCGGGACGCGGAAGTCCCCCGCTGTGAGGTTGAGAAAGAGGC
TCCCACTGGCTCCGAGCCT
    -A        C TA       GG      C    ----     AC CG
AGGC  GGAGCCGG T  GAGCCTC  TTTCTC ACCT    GCGGG  G  G
||||  |||||||| |  |||||||  |||||| ||||    |||||  |  A
TCCG  CCTCGGTC A  CTCGGAG  AAAGAG TGGA    CGCCC  C  A
    AG        - CC       --      T    GTGT     CC TG
```

```
23)TTTGCTAGTGGTGGGTCACCCTCTGTTGCTAGGGAAACGGTTCCCTAGCAACAGAACGCCACTATTTGCTAG
  T  T        GTCA CC              A
TT GC AGTGGTGG    C  TCTGTTGCTAGGGAA C
|| || ||||||||    |  |||||||||||||||
GA CG TTATCACC    G  AGACAACGATCCCTT G
  T  T        ---- CA              G
```

```
24)GTGTCTCCTTGTCCATCTGAAGAAGATGCTGCTATGTGGAAGCACATCCTTGAGATCCACAAGGAGACAC
            CC    G   A GA    G
GTGTCTCCTTGT  ATCT AAG A  TGCT CTAT
||||||||||||  |||| ||| |  |||| |||
CACAGAGGAACA  TAGA TTC T  ACGA GGTG
            CC    G   C AC    A
```

```
25)AGAGGGTGGGGAATTGGGGTTTCCTGCCATGGGGAGGGAGAGGATTGAGCTCCAAGGTAAACAGACCCCAAACCCTTCCC
TCC
A     T    AA         -CC    A     AGGGAG
 GAGGG GGGG  TTGGGGTTT   TGCC TGGGG      A
 ||||| ||||  |||||||||   |||| |||||      G
 CTCCC TCCC  AACCCCAGA   ATGG ACCTC      G
C     T    -A         CAA    A     GAGTTA
```

```
26)GGTACCAGCAGGTGCAGGTGTGTGACTGCCTGTGAGCCTGTCACTGCATCTGCACCTGCTTACT
    CC              -     T C
GGTA  AGCAGGTGCAGGTGT GTGAC G CTG
||||  ||||||||||||||| ||||| | ||T
TCAT  TCGTCCACGTCTACG CACTG C GAG
    --              T     T C
```

```
27)TGCCATCTTGGCTATGACCCAAGTCTCTCCCAGGGTTTCAGGTTGACTTGGCACCTTGAAGAGTGGAGGCTTGGCCCCCA
GGATGAGG
 GC        CTAT AC          C ----      T
T  CATCTTGG    G  CCAAGTCTCT C    CAGGGT TCAGGTT
|  ||||||||    |  |||||||||| |    |||||| ||||||
G  GTAGGACC    C  GGTTCGGAGG G    GTTCCA GGTTCAG
 GA        ---- CC          T AGAA      C
```

```
28)AATCTCAGGACAGTGGCAGGACTGGGCATGCGGATGCCTTGGACCTCTCAAGGTCACTGGCCCCACTTCCCAGTCTTGGC
TAGTCCTGGGA
AA         AGT G          C TGC  AT   TTG     C
  TCTCAGGAC   G CAGGACTGGG A   GG  GCC   GACCT T
  |||||||||   | |||||||||| |   ||  |||   |||||
  AGGGTCCTG   C GTTCTGACCC T   CC  CGG   CTGGA C
--         -AT G          T CAC  --   TCA     A
```

```
29)TTGGTGCGTGGTTCTGGCTTAGTTGTGCTGACTCAGTCTTAGGCCTGGGGACAGTGGGTTAGTCACAAGTTGGTAGAACT
ACGCATCTC
TT              G TTAG TGT         GTCTTAGG
  GGTGCGTGGTTCTG C    T   GCTGACTCA        C
  |||||||||||||| |    |   |||||||||        C
  CTACGCATCAAGAT G    A   TGATTGGGT        T
CT              G TTGA CAC         GACAGGGG
```

```
30)TCTGTCTCTCTCCTGTGCTTTTCTACCTCCCCGGGAAGGTGGTAAATGTTAGAACAGCATCTCCCTGAGGGTAAGAAAAG
TCAGACTAGTGATAGC
T     T  CTC   T        AC T  C    A     GTAAATG
 CTGTC CT   CTG GCTTTTCT  C CC CGGG AGGTG       T
 ||||| ||   ||| ||||||||  | || |||| |||||
 GATAG GA   GAC TGAAAAGA  G GG GTCC TCTAC       T
C     T  TCA   -        AT -  A    C     GACAAGA
```

```
31)CTCTCATGGCAGGAACTCTGCTTAAATCCTGACTAGACTCCCAGGTGCCCACAGAACATGGGAGTAGCCTGGTCAGTTAC
AGAGCCTGTTTGAGGG
      T      AA     CT   ATC        ---       G  G C
CTCTCA GGCAGG  CTCTG  TAA   CTGACTAG   ACTCCCA GT C C
|||||| ||||||  |||||  |||   ||||||||   ||||||| || | A
GGGAGT TTGTCC  GAGAC  ATT   GACTGGTC   TGAGGGT CA G C
      -      --     --   ---        CGA       A  A A
```

```
32)GGTCCCCGAGGGGATACAGGGCCGTGCAGCCTCTTGCCTGAGGGTTTGCATGTCTGCCTGGATCCCCCGGGCTCC
  TC    A      A    -- C       C    TG
GG  CCCG GGGGAT CAGG  G CGTGCAG CTCT  C
||  |||| |||||| ||||  | ||||||| ||||
CC  GGGC CCCCTA GTCC  C GTACGTT GGGA  C
  TC    -      G    GT T       T    GT
```

```
33)GCCTGGGGGAGGTGGAGCTCTGAGAATTCCACGCCCTGTGGGGGGTGGAGTTCTCATGGAGCCCCGCCCCTGGGT
         AGG   A    --            G   T
GCCTGGGGG   TGG GCTC  TGAGAATTCCAC CCC G
|||||||||   ||| ||||  |||||||||||| |||
TGGGTCCCC   GCC CGAG  ACTCTTGAGGTG GGG T
         ---   C    GT            G   G
```

```
34)GCCATGGGCCTTAATATGCTGTGAATCTCCCTCAGCTGTCACAGCAGGAGGGGCCATCATAACATCCCAAAGGGTCCAGG
GC
   A        --AAT   C     ATC      -A
GCC TGGGCCTT     ATG TGTGA   TCCCTC  GCTGT
||| ||||||||     ||| |||||   ||||||  |||||C
CGG ACCTGGGA     TAC ATACT   GGGGAG  CGACA
   G        AACCC   A     ACC      GA
```

```
35)GAGCTGCCTGCCCCTGGCCTCCTGGGAAGGTGGCTTCTTGCCGAGCCTCCAGGGTGGCCCAGGTAGGTAGGCC
 AG         CCT G C       AAGG    T
G  CTGCCTGCC   G C TCCTGGG    TGGC T
|  |||||||||   | | |||||||    |||| C
C  GATGGATGG   C G GGGACCT    GCCG T
 CG         ACC G T       CCGA    T
```

```
36)CTGGGGGTCCCAGGGGCTGGAGGCCTGCCTGGTTAGCCTCTGTTTCCCAGACATTGACTCGAGGCGCCTCCAGTCCTCTC
AACCCCCTG
 T      CCCA            CTGC T      CC    T
C GGGGGT    GGGGCTGGAGGC    C GGTTAG  TCTG T
| ||||||    ||||||||||||    | ||||||  |||| T
G CCCCCA    TCCTGACCTCCG    G TCAGTT  AGAC C
 T      ACTC            CGGA C      AC    C
```

```
37)ACAGTGTGTACTAAGCAGGTGCTCAGCAAGTGCAGTGTGTACTTAGCAGGCACTTAGCAGGCGCACAGTGT
   G     A  AAGC       CAGC
ACA TGTGT CT    AGGTGCT    AAGTGCAG
||| ||||| ||    |||||||    |||||||T
TGT ACACG GG    TTCACGG    TTCATGTG
   G     C  ACGA       ACGA
```

```
38)GTGTGCTCTGCAGGCGCTTAGCAGGTGCACAGTGTGTATTTAGCAGGCACTCAGCTGACACACAGTGTGTATTTAGCAGG
CGCTCAGCAGGCACAG
G      CTGCA       AGC         G     ATTTAGCAGG
 TGTGCT     GGCGCTT   AGGTGCACA TGTGT          C
 ||||||     |||||||   ||||||||| |||||
 ACACGG     TCGCGGA   TTTATGTGT ACACA          A
G      ACGAC       CGA         G     CAGTCGACTC
```

```
39)GTTTACTGAGGAACTCTGGTGGACCAGCCATGCAGCGAGGGGTCAGCTCAGCTCTTCTCATCAGGTCCTCAGTAAAC
            AC       -  CC   CAT  AGCGA
GTTTACTGAGGA  TCTGGTG GA  AGC   GC     G
||||||||||||  ||||||| ||  |||   ||
CAAATGACTCCT  GGACTAC CT  TCG   CG     G
            --       T  TC   ACT  ACTGG
```

```
40)CCCGGGGCCCTGAAGCCATCCCTGTCCCCTGTAGAGGGGATGCTCTCGTCCTACTTCCCAGCTAGATAAAAGAGTCGCTC
CTCCAGGGCTGAGGTCGGGCTCCCTTGTCCCAGG
  C     CCT A    ATC   GTC     TA      AT     CGTCCTACTTC
CC GGGGC   G AGCC   CCT   CCCTG  GAGGGG  GCTCT           C
|| |||||   | ||||   |||   |||||  ||||||  |||||
GG CCCTG   C TCGG   GGA   GGGAC  CTCCTC  TGAGA           C
  A     TTC C    GCT   GTC     --      GC     AAATAGATCGA
```

```
41)GCTGGCGGGGACCCCGCACTTGGGCGGGCGTGGATCGGAGGCCTCCGCCTGTCCGCCGAGGGCGTTCCCGTCGTC
 C          C  GCACT          T  A
G TGGCGGGGAC CC     TGGGCGGGCG GG TCG
| |||||||||| ||     |||||||||| || ||G
C GCTGCCCTTG GG     GCCTGTCCGC TC GGA
 T          C  GAGCC          C  C


42)TCTGCCCAGTGGGCACTGCTCTGGGCACATCAGGCGCTGTCCTCTGCACAGTCCAGTGCCCACTGAGGAAGGCAGG
      -----            CT   G   CATCAGG
TCTGCC     CAGTGGGCACTG  CTG GCA       C
||||||     ||||||||||||  ||| |||       G
GGACGG     GTCACCCGTGAC  GAC CGT       C
      AAGGA            CT   A   CTCCTGT


43)TGCCCAGTGGGCACTGCTCTGGGCACATCAGGCGCTGTCCTCTGCACAGTCCAGTGCCCACTGAGGA
 G C            CT   G   CATCAGG
T C CAGTGGGCACTG  CTG GCA       C
| | ||||||||||||  ||| |||       G
A G GTCACCCGTGAC  GAC CGT       C
 G A            CT   A   CTCCTGT


44)GGGCCACCGTTGGGAACCAAGACGTGTCCTGCCCAGGCGCACGGCATGTCCCCTGCATTTGGTTCTCAGGAAGGCCC
     AC G           --------        C   C
GGGCC  C TTGGGAACCAA        GACGTGTC TGC CA
|||||  | |||||||||||        |||||||| ||| |
CCCGG  G GACTCTTGGTT        CTGTACGG ACG GG
     AA -           TACGTCCC        C   C


45)TGCCCTAGGGGACCTAGGGACTTCCTTCTGTGCCTGACCTCTAGAGGTGGCATGGAAGAAGAGTCCCACAGCAGACCCTG
GGGCT
T         --GA CTA       -C          TG
 GCCCTAGGG    C   GGGACTT  CTTCTGTGCC  ACCTCT
 |||||||||    |   |||||||  ||||||||||  |||||
 CGGGGTCCC    G   CCCTGAG  GAAGGTACGG  TGGAGA
T         AGAC ACA       AA          --


46)GCTCCCACTGGACCTAAGGCACTGTCTCTCCTATTCTCTGTGGATCAGGGGCTGCCTTGGGTTCAGAGGACAT
 CTC  A              CT      CCTATTC
G   CC CTGGACCTAAGGCA  GTCTCT       T
|   || ||||||||||||||  ||||||       C
T   GG GACTTGGGTTCCGT  CGGGGA       T
 ACA  A              --      CTAGGTG


47)CGCGCCGCCTTCTCTTGTTGCACCCAACATGGCGGCCATCGTGGGAGCCGCGTGGAGAGAGCGATGCG
    C   C      -   T  A    AC
CGCG CGC TTCTCT TGT GC CCCA  ATGGC
|||| ||| |||||| ||| || ||||  |||||G
GCGT GCG GAGAGG GCG CG GGGT  TACCG
    A   A      T   C  A    GC
```

```
48)ACCTGCTGACCACCACTTTGTGTCATCACTGTTTGGATGACACAAAGGGTCGTTTGAAGG
A   G T   C   A             ACT
 CCT C GAC ACC CTTTGTGTCATC   G
 ||| | ||| ||| ||||||||||||
 GGA G TTG TGG GAAACACAGTAG   T
-   A T   C   -             GTT
```

```
49)CCGGGTGTGTGTTGTGCGTGCGTGCACTTGCACGGCATGTGCGTGCATGTTGACATGGCAGCCGG
    G   GTGT GT             T
CCGG TGT    T  GCGTGCGTGCAC TGCA
|||| |||    |  |||||||||||| |||C
GGCC ACG    A  TGTACGTGCGTG ACGG
    G   GTAC GT             T
```
