# Supplementary material for: Prediction of novel precursor miRNAs using a context-sensitive hidden Markov model (CSHMM)
Source: BMC Bioinformatics. 2010 Jan 18;11(Suppl 1):S29. doi: 10.1186/1471-2105-11-S1-S29 (PMC3009500; doi:10.1186/1471-2105-11-S1-S29)
Supplement: Additional file 5 — Novel miRNAs from Small RNA sequence analysis. The sRNAs are grouped into their respective IsomiR families as and when present. The ones highlighted in blue are the representative novel miRNAs, identified on the basis of highest frequency within the family. [file 1471-2105-11-S1-S29-S5.pdf]

Small RNA analysis : Common sRNAs predicted to be falling within the hairpin by the three prediction programs.

S.No	Small RNA sequence	Length	Frequency	Chromosomal location	CSHMM*	CID-miRNA*	MIRDEEP
1	TATACAGGGGGAGACTCTCAT	21	2	chr14.NT_026437.[82473123..82540116] (37169..37248)(+)	0.256	-0.595697	2.4
2	ATATACAGGGGGAGACTCTCAT	22	21				
3	ATATACAGGGGGAGACTCTCATT	23	4				
4	ATATACAGGGGGAGACTCTCA	21	4				
5	ATATACAGGGGGAGACTCTCATTT	24	3				
6	TATACAGGGGGAGACTCTTA	20	1	chr14.NT_026437.[82473123..82540116] (35947..36028)(+)	0.253	-0.597301	2.3
7	ATATACAGGGGGAGACTCTTA	21	27				
8	TATACAGGGGGAGACTCTTAT	21	1				
9	TATACAGGGGGAGACTCTTATT	22	1				
10	ATATACAGGGGGAGACTCTTAT	22	181				
11	ATATACAGGGGGAGACTCTTATT	23	27				
12	AATATACAGGGGGAGACTCTTAT	23	7				
13	ATATACAGGGGGAGACTCTTATTT	24	12				
14	TAAAAACCGTGACTACTTC	19	1	chr6.NT_025741[12262885..12360950] (4039..4122)(-)	0.304	-0.533041	2.5
15	TAAAAACCGTGACTACTTCT	20	2				
16	TAAAAACCGTGACTACTTCTG	21	2				
17	CCTCCCACTGCAGAGCCTGGGGA	23	1	chr10.NT_077569 [521740..607790] (35288..35409)(+)	0.271	-0.49226	1.5
18	CGGTTTGAGGCTACAGTGAGAT	22	7	chr11.NT_008984.[5728386..5729897] (75..191)(-)	0.281	-0.470894	1.1

- **the blue colour signifies the representative mirna (having the highest frequency among its isomiRs)**
- *** signifies the score assigned to the precursor harboring the miRNA and its isomiRs.**
