# Supplementary material for: Prediction of novel precursor miRNAs using a context-sensitive hidden Markov model (CSHMM)
Source: BMC Bioinformatics. 2010 Jan 18;11(Suppl 1):S29. doi: 10.1186/1471-2105-11-S1-S29 (PMC3009500; doi:10.1186/1471-2105-11-S1-S29)
Supplement: Additional file 6 — Secondary structures of the 5 representative novel miRNA from the sRNA sequence data. [file 1471-2105-11-S1-S29-S6.pdf]

```
(1)srnaseq:atatacagggggagactctcat Length:22 Freq:21 chromosomal_pos:
   chr14.NT_026437.[82473123..82540116] (37169..37248)(+)
   ggtacttaaagagaggataccctttgtatgttcacttgattaatggcgaatatacagggggagactctcatttgcgtatc
   0.2555
       t    -     ga a              a ttga
  ggtac taaa gagag  t ccctttgtatgttc c    t
  ||||| |||| |||||  | |||||||||||||| |
  ctatg gttt ctctc  a gggggacatataag g    t
       c    a     ag -              c gtaa

  MiPred Score: Real 85.3%


(2)srnaseq:atatacagggggagactcttat Length:22 Freq:181 chromosomal_pos:
   chr14.NT_026437.[82473123..82540116] (35947..36028)(+)
   tggtacttgaagagaggataccctttgtatgttcacttgattaatggcgaatatacagggggagactcttatttgcgtat
ca
   0.2533
        t    g  a  ata              a ttga
  tggtac tgaa ag gg   ccctttgtatgttc c    t
  |||||| |||| || ||   |||||||||||||| |
  actatg gttt tt tc   gggggacatataag g    t
        c    a  c  aga              c gtaa

  MiPred Score: Real 85.1%


(3)srnaseq:taaaaaccgtgactacttctg Length:21 Freq:2 chromosomal_pos:
   chr6.NT_025741.[12262885..12360950](4039..4122)(-)
   tattgggtgggtgcaaaagtaattgcggtttttgctattagtttcaatggtaaaaaccgtgactacttctgcaccaacct
agta
   0.3040
          g      a     a                 ag
  tattgggt ggtgca aagta ttgcggtttttgctatt  t
  |||||||| |||||| ||||| |||||||||||||||||
  atgatcca ccacgt ttcat agtgccaaaaatggtaa  t
          a      c     c                 ct
  MiPred Score: Real 83.7%


(4)srnaseq:cctcccactgcagagcctgggga Length:23 Freq:1 chromosomal_pos:
   chr10.NT_077569.[521740..607790] (35288..35409)(+)
   tggtgacccacacttttgagatgcctgttccgggcatcacctcccactgcagagcctggggagccggacagctcccttcc
caggctctgcagtgggaactgatgcctggaacagttcctgca
   0.2709
    gt acccacactttt   atgc               cc                      cc   c
  tg  g           gag    ctgttccgggcatca  tcccactgcagagcctggggag  gga a
  ||  |           |||    |||||||||||||||  ||||||||||||||||||||||  |||
  ac  c           ctt    gacaaggtccgtagt  agggtgacgtctcggacccttc  cct g
    gt ------------   ----               ca                      --   c

  MiPred Score: Real 73.4%
```

```
(5)srnaseq:cggtttgaggctacagtgagat Length:22 Freq:7 chromosomal_pos:
chr11.NT_008984.[5728386..5729897] (75..191)(-)
  ctactctggaggctaaggcaggagaaacacttgagcccagcggtttgaggctacagtgagatgtgatcctgccacatctca
ctgtagcctcgaacccctgggctcaagtgattcagc
  0.2815
  ctactctggag   aaggcagga   a             c                        at
             gct         gaa cacttgagcccag ggtttgaggctacagtgagatgtg  c
             |||         ||| ||||||||||||| ||||||||||||||||||||||||  c
             cga         ctt gtgaactcgggtc ccaagctccgatgtcactctacac  t
  -----------   ---------   a             c                        cg
  MiPred Score: Real 76.4%
```
